# IQCODE‐Brief Version in Stroke Patients

**DOI:** 10.1002/brb3.71649

**Published:** 2026-08-02

**Authors:** Mine Sezgin, Edis Hacılar, Sevda Özel‐Yıldız, Ozan Dörtkol, Esme Ekizoğlu, Nilüfer Yeşilot

**Affiliations:** ^1^ Department of Neurology İstanbul Faculty of Medicine İstanbul University İstanbul Türkiye; ^2^ Department of Biostatistics, Institute of Graduate Studies in Health Sciences İstanbul University İstanbul Türkiye; ^3^ Department of Biostatistics İstanbul Faculty of Medicine, İstanbul University İstanbul Türkiye

**Keywords:** cognition, dementia screening, IQCODE brief version, post‐stroke cognitive impairment, stroke

## Abstract

**Introduction:**

Post‐stroke cognitive impairment is frequently seen after stroke. We aimed to examine the psychometric properties, factorial structure, and screening utility of the IQCODE brief version in stroke patients using an informant‐based approach to cognitive impairment screening.

**Methods:**

Patients aged older than 18 years with ischemic or hemorrhagic stroke were included. Within one week of completing the MoCA test, each patient's informant (spouse, caregiver, etc.) was contacted by phone, and the IQCODE brief version was administered. Cronbach's alpha, test‐retest analysis, exploratory factor analysis (EFA), and confirmatory factor analysis (CFA) were performed to assess the reliability and construct validity of the IQCODE brief version.

**Results:**

A total of 179 patients with stroke (mean age: 58.6 ± 13.7 years) were included, 18 (10%) of them had hemorrhagic stroke. The IQCODE brief version demonstrated excellent internal consistency (Cronbach's alpha = 0.94). Factor analysis suggested a preliminary four‐factor structure, explaining 76.5% of the total variance. CFA confirmed a good model fit (RMSEA = 0.07, CFI = 0.973, TLI = 0.964). Review of the factor loadings resulted in the exclusion of three items from the initial model. ROC analysis for the 16‐item version yielded an area under the curve (AUC) of 0.771 (95% CI, 0.70 – 0.84), with an optimal cut‐off score of 48.5 (sensitivity: 72.4%; specificity: 71.1%). A refined 13‐item version demonstrated improved specificity (76%) without compromising sensitivity.

**Conclusions:**

Both the 16‐item and the proposed 13‐item IQCODE brief versions demonstrated promising preliminary psychometric properties, and they may serve as effective screening tools. Their feasibility for telephone administration and strong psychometric properties support their use in routine clinical practice and remote screening.

## Graphical abstract

Graphical abstract illustrating the psychometric evaluation of the IQCODE brief version in patients with stroke. Factor analysis supported refinement from 16 to 13 items, with the refined version demonstrating improved specificity without compromising sensitivity. Both versions showed promising preliminary psychometric properties and may be useful for routine clinical practice and remote screening.

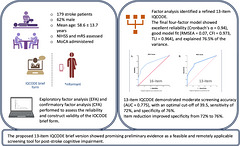

## 1 Introduction

Post‐stroke cognitive impairment (PSCI) is common after both ischemic and hemorrhagic stroke; however, it is frequently underrecognized (Donnellan and Werring 2020; Gottesman and Hillis 2010; Planton et al. 2017). While definitions and subtypes of PSCI and and post‐stroke dementia may vary depending on the duration after the index stroke and other preexisting comorbidities, the prevalence of PSCI was reported approximately up to 40% (Rost et al. 2022). Accurate diagnosis of PSCI is clinically important because cognitive impairment influences functional outcome, long‐term independence, and also treatment adherence. However, diagnosis of PSCI is usually time‐consuming, and may require a multidisciplinary approach. As a result of the ambiguity of diagnostic procedures and lack of awareness among physicians, a substantial proportion of these patients remain underdiagnosed (El Husseini et al. 2023). Additionally, cognitive problems in patients with stroke are intricate, and often, they are not the primary focus of inpatient and outpatient visits after stroke (Abzhandadze et al. 2021; Brainin et al. 2015).

Although detailed neuropsychological evaluation can help overcome diagnostic challenges in PSCI, it requires specialized training for both application and interpretation (Harvey 2012). Screening tests for PSCI are required for accurate diagnosis, as they help reduce the target patient population who may need more comprehensive assessments of all cognitive domains. Various tests have been proposed as candidates for screening and diagnosing PSCI, such as the Montreal Cognitive Assessment test (MoCA) and the Mini‐Mental State Examination (MMSE), which are the most used paper‐pencil tests (Burton and Tyson 2015; Quinn et al. 2018). Among the screening tests, MoCA has demonstrated better sensitivity and specificity in stroke patients (Nasreddine et al. 2005; Suda et al. 2020). Nonetheless, due to patient overload and staff shortages, even the MoCA test might be unsuitable for most clinicians and other healthcare providers, especially in developing countries. Shorter versions of screening tests are available and are suggested in routine clinical practice (Hachinski et al. 2006), but evidence is currently limited (Quinn et al. 2018). In addition, there are particular difficulties when evaluating stroke patients with PSCI, such as co‐existing aphasia and pre‐stroke cognitive impairment.

Informant‐based tests may overcome these barriers because of their convenience and accessibility and may contribute to understanding PSCI. The Informant Questionnaire on Cognitive Decline in the Elderly (IQCODE) is a widely known screening test, and it is used extensively in different languages (Fuh et al. 1995). Its sensitivity and specificity for screening dementia were shown in a comprehensive systematic review (Taylor‐Rowan et al. 2023). Additionally, mild cognitive impairment could be detected with IQCODE (sensitivity 97.9%; specificity 71.4%) (Abd Razak et al. 2019). One distinctive feature of the IQCODE is that the educational levels of patients and previous occupations do not significantly influence IQCODE results (Carrabba et al. 2015; Christensen and Jorm 1992). While the original version includes 26 items, the IQCODE‐brief version contains 16 items (Jorm 1994, 2004). The IQCODE brief version's ability to screen dementia was also proven to have 88% sensitivity and 80% specificity in different patient populations (Taylor‐Rowan et al. 2023). Even though the IQCODE is primarily used to detect pre‐stroke cognitive impairments, it has also been shown to play an important role in detecting post‐stroke cognitive deficits in patients with cerebrovascular disease (McGovern et al. 2016). However, there is limited evidence on the factorial structure and optimal cut‐off values of the IQCODE brief version for detecting PSCI in stroke populations.

In this study, we aimed to examine the psychometric properties, factorial structure, and screening utility of the IQCODE brief version in stroke patients using an informant‐based assessment approach to cognitive impairment screening. A secondary exploratory objective was to investigate whether the removal of poorly performing items could improve the psychometric performance of the scale. In addition, we aimed to determine an optimal cut‐off value for the IQCODE‐ brief version, using the MoCA‐based cognitive classification as a reference screening approach.

## 2 Methods

### 2.1 Participants

Patients with stroke who were followed at the Istanbul University Medical Faculty Neurology Clinic were invited to participate in the study. We included patients over the age of 18, diagnosed with ischemic or hemorrhagic stroke, who had survived at least three months since their last stroke and agreed to participate in the study with written consent. Patients with prior neurodegenerative disease, with a Modified Rankin Scale (mRS) of 4 and 5, or with a history of severe head trauma were excluded from the study. Clinical and demographic data were recorded for index stroke events. A vascular neurologist (MS or NY) examined each patient and performed the National Institutes of Health Stroke Scale (NIHSS) assessment. Functional status was assessed using the mRS and the MoCA test. All participants underwent MoCA assessment during an outpatient follow‐up visit conducted at least three months after the index stroke. Within one week of MoCA administration, informants were interviewed by telephone using the brief version of the IQCODE. Informants were defined as the patient's spouse, caretaker, or first‐degree relative living with the patient. The MoCA test was administered to screen for cognitive impairment and was used as a reference screening tool for comparison. All participants or their legal authorized representatives provided written informed consent, and the local Ethics Committee of Istanbul University approved the study (protocol number: 2023/315).

### 2.2 Statistical Analysis

All statistical analyses were conducted with SPSS version 24 and Jamovi version 2.3.26.0. Descriptive statistics were reported as means, medians, standard deviations, and interquartile ranges when suitable. Missing data were handled with an imputation method, specifically by replacing mean values with numeric continuous variables. Internal consistency was estimated with Cronbach's alpha test. Exploratory factor analysis (EFA) and confirmatory factor analysis (CFA) were performed to assess the construct validity and reliability of the brief version of the IQCODE. Before conducting EFA sampling adequacy was confirmed with the Kaiser Meyer Olkin (KMO). Additionally, the Tucker‐Lewis index (TLI) and Bartlett's test of sphericity results were evaluated for the model's fit and the adequacy of the sample size (Tucker and Lewis 1973). The number of factors was determined according to eigenvalues (>1), total explained variance, and the scree plot. Minimum residual extraction with oblimin rotation was used because the factors were correlated in the EFA (*r* = 0.43). Variables with factor loadings less than 0.40 were sequentially removed, and EFA was repeated after each removal to ensure the stability of the factor structure. CFA was conducted using the same dataset and was re‐performed with the same dataset after the aforementioned removals. The root mean square error of approximation (RMSEA) values less than 0.05 and 0.05–0.08 were determined to be perfect and good fit, respectively. A comparative fit index (CFI) between 0.97 and 1 was a perfect fit, and CFI values between 0.95 and 0.97 were a good fit. Cronbach's alpha was additionally calculated for each factor in the final model, addressing the scale multidimensionality. Test‐retest reliability was assessed with correlation analysis and the Bland‐Altman graphical approach. The IQCODE brief version was applied to the same subgroup of patients one month apart.

Receiver operating characteristic (ROC) analysis was performed separately for the 16‐item and 13‐item IQCODE bried versions to determine cut‐off values to screen for cognitive issues. For both versions of the IQCODE, total scores were calculated by summing the responses to all questionnaire items. MoCA results were used to dichotomize patients, and the cut‐off value was set at 25 points, which was used for the outcome variable in the ROC analysis. The Youden index (*J* = sensitivity + specificity − 1) was calculated to find cut‐off values for total IQCODE brief version scores. The Spearman correlation coefficient was used to analyze the association between the IQCODE brief version and several clinical features. Categorical data were compared with the chi‐square test, and the t‐test or Mann‐Whitney U test was used after normality assessments for continuous variables.

The sample size for CFA was calculated using the rule *n* = number of items × 10; thus, at least 16 × 10 = 160 participants were required (Boeteng et al. 2018). For the ROC analysis sample size calculation, area under the curve (AUC): 0.85; estimated disease percentage 0.40, confidence interval width *W* = 0.125, and confidence interval 0.95 were taken; the total sample size was found to be 173. Since a sample size of at least 160 patients was calculated for CFA and 173 patients for ROC, 179 stroke patients were included in the study, considering the possibility of dropouts.

Finally, MoCA tests and clinical evaluations were performed by MS, NY, and EE, while IQCODE brief version telephone interviews were conducted by EH, who was blinded to the clinical assessments.

## 3 Results

One hundred and seventy‐nine patients with stroke participated in the study. There were 113 (63.1%) males, and the mean age was 58.6 (±13.7) years. The majority of the participants had an ischemic stroke, only 18 (10%) patients had a hemorrhagic stroke. Additional clinical characteristics are given in Table 1. Forty‐five patients (25%) had MoCA scores of more than 24 points. All the informants resided with the patients. Missing data rates were 1% for age (*n* = 2), 4% (*n* = 8) for years of education, and 1% (*n* = 2) for the NIHSS score at assessment. No missing data were observed for IQCODE or MoCA scores.

**TABLE 1:** Demographical and clinical features of stroke patients.

	Ischemic Stroke (*n* = 161)	Hemorrhagic stroke (*n* = 18)	*p*
Sex (male, *n*, (%))	100, (62.1)	13, (72.2)	0.39^ǂ^
Age (year, mean (SD); median IQR)	58.5 (±13.5); 59 (51–68)	59.7 (±15.7); 59.5 (52–70)	0.60^#^
Education (years, mean (SD); median IQR)	8.6 (±4.4); 8 (5–12)	9.6 (±5.1); 12 (5–15)	0.40^#^
Hypertension, *n*, (%)	122, (75.8)	13, (72.2)	0.71^ǂ^
Diabetes, *n*, (%)	55, (34.2)	2, (11.1)	0.09^ǂ^
Atrial fibrillation, *n*, (%)	23, (14.3)	1, (5.6)	0.70^ǂ^
Lesion localization, *n*, (%)			
Supratentorial Right Bilateral	96, (59.6) 59, (36.6) 21, (13.1)	16, (88.9) 7, (38.9) 3, (16.7)	0.053^ǂ^ 0.87
NIHSS at index stroke (mean (SD); median IQR)	4.9 (±5.5); 3 (1–6)	5.4 (±3); 5 (3–7.5)	0.07^#^
mRS ≥ 3 at index stroke *n*, (%)	95, (60)	13, (72.2)	0.20^ǂ^
Duration after stroke (month: mean (SD); median IQR)	33.8 (±33.4); 21 (7–51.5)	35.6 (±26.2); 30.5 (11.7–59.7)	0.36^#^
NIHSS (at last control: mean (SD); median IQR)	1 (±1.5); 1 (0‐2)	1.7 (±1.3); 2 (1–2.5)	0.009^#^
mRS ≥ 3 (at last control)	11, (6.8)	3, (16)	0.14^ǂ^
Total MoCA score (mean (SD); median IQR)	19.8 (±6); 21 (16–25)	19.1(±5.3); 21 (17–22.2)	0.48^#^
Total IQCODE brief form score (mean (SD); median IQR	51.6 (±5.6); 50 (48–52)	54.2 (±8.3); 51 (48–57.5)	0.31^#^

^Note: ǂ^ Chi‐square test.

^Note: #^ Mann‐Whitney U test.

Abbreviations: N, number; SD, standard deviation; NIHSS, National Institutes of Health Stroke Scale; mRS, modified Rankin scale; MoCA, Montreal Cognitive Assessment test, IQCODE, The Informant Questionnaire on Cognitive Decline in the Elderly.

### 3.1 Findings for the 16‐Item IQCODE Brief Version

The internal consistency test resulted in a Cronbach's alpha value of 0.95 when all 16 items in the questionnaire were included. EFA indicated an adequate sample size and suitability for factor analysis (KMO = 0.91 and Bartlett's sphericity test, *p* < 0.001 (*x* ^2^ = 2428) in the initial model (Model 1). The final model was derived after factor loadings were examined, and extraction was performed accordingly. The factor loadings of Model 1 and the final model are given in Table 2. Item 1, Item 8 and Item 9 were sequentially removed based on the factor loadings, and factor loads were re‐calculated at each step. Items 1, 8, and 9 demonstrated weaker or unstable factor loadings and were therefore removed to improve factorial coherence and model fit. The removal of these items was not intended to imply a lack of clinical importance. Item 1 reflects autobiographical memory, whereas items 8 and 9 are related to the use of machines and technological devices. These items may be less sensitive to PSCI.

**TABLE 2:** The factor loadings for different models of IQCODE brief version.

	Model 1* (16 items)			Final model** (13 items)			
Variables	Factor 1	Factor 2	Factor 3	Factor 1	Factor 2	Factor 3	Factor 4
Item 1	0.336	—	—	—	—	—	—
Item 2	0.538	—	—	—	—	—	0.530
Item 3	0.441	—	—	—	—	—	0.938
Item 4	—	—	1.031	—	—	1.017	—
Item 5	—	—	0.690	—	—	0.670	—
Item 6	—	0.916	—	—	0.964	—	—
Item 7	—	0.813	—	—	0.809	—	—
Item 8	0.411	—	0.374	—	—	—	—
Item 9	0.571	—	—	—	—	—	—
Item 10	0.690	—	—	0.601	—	—	—
Item 11	0.888	—	—	0.831	—	—	—
Item 12	0.743	—	—	0.715	—	—	—
Item 13	0.830	—	—	0.845	—	—	—
Item 14	0.883	—	—	0.861	—	—	—
Item 15	0.883	—	—	0.913	—	—	—
Item 16	0.772	—	—	0.658	—	—	—

*Note*: Standardized pattern matrix coefficients obtained using the minimum residual extraction method with oblimin rotation are presented.

*KMO = 0.91 and Bartlett's Sphericity test *p* < 0.001 (*χ* ^2^ = 2428).

** KMO = 0.91 and Bartlett's Sphericity test *p* < 0.001 (*χ* ^2^ = 1943).

### 3.2 Findings for the 13‐Item IQCODE Brief Version

**FIGURE 1:**
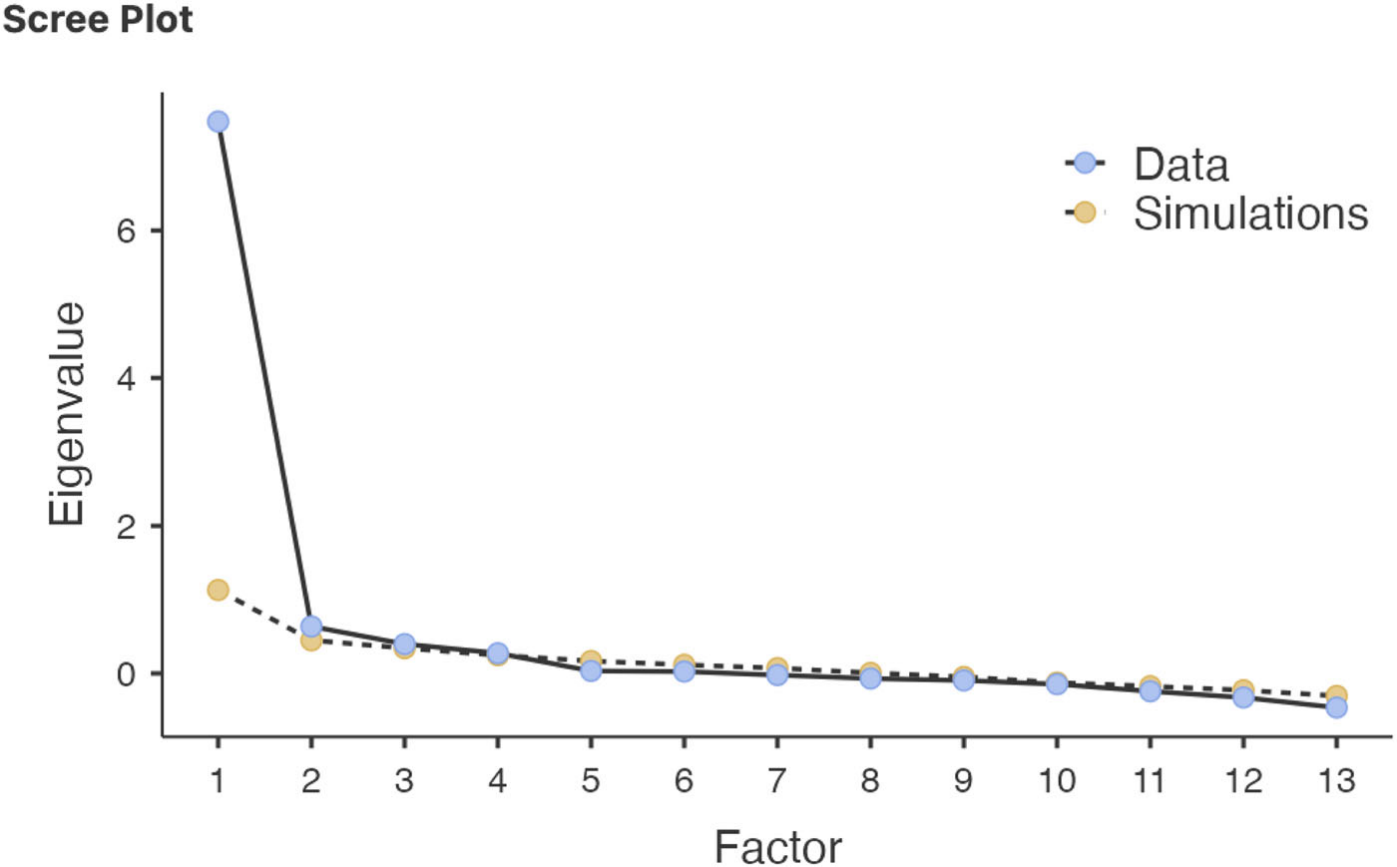
Scree plot of final model.

After three items were excluded, the remaining test was renamed the 13‐item IQCODE Brief Version. The internal consistency was re‐evaluated for the 13‐item IQCODE Brief Version, and the new Cronbach's alpha value was 0.94. The factor loadings of the final model are given in Table 2. The four‐factor structure was derived based on the scree plot and a careful examination of factor loadings (Figure 1). Afterward, CFA analysis with four‐factors revealed RMSEA = 0.07 (0.05–0.09), CFI = 0.973, and TLI = 0.964, which were compatible with a good fit and the cumulative variance explained by the 4 factor was 76.5%. Secondary factor loadings and communality values of the questionnaire items are shown in (Supplementary Table 2 and 3). Secondary factor loadings support a relatively stable multidimensional factor structure with partially correlated cognitive domains. Inter‐factor correlations ranged from 0.417 to 0.632, supporting the use of oblique rotation and indicating moderate associations among the extracted factors (Supplementary Table 4). These findings are consistent with the overlapping nature of cognitive domains in PSCI. Although the factors demonstrated clinical interpretability, the moderate inter‐factor correlations suggest that these dimensions should not be considered entirely independent neuropsychological constructs. Cronbach's alpha coefficients for the extracted factors ranged from 0.803 to 0.938, indicating good‐to‐excellent internal consistency. However, reliability estimates for factors containing only two items should be interpreted cautiously.

**FIGURE 2:**
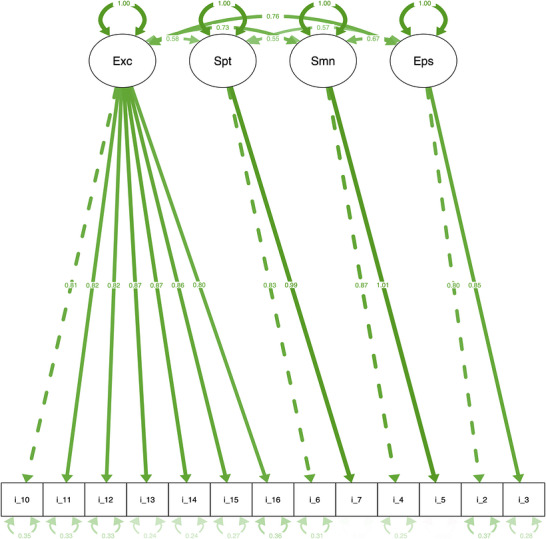
Path diagram of the confirmatory factor analysis. Abbreviations: I, item; Exc, executive and complex daily functioning–related items (Factor 1); Spt, visuospatial orientation and spatially related activities (Factor 2); Smn, semantic knowledge and language‐related activities (Factor 3); Eps, episodic memory–related activities (Factor 4).

The identified factors were evaluated and named according to relevant cognitive domains to facilitate interpretation. The four factors of the final model were labeled according to their item content and their corresponding clinical relevance. Factor 1 appears to primarily reflect executive and complex daily functioning, Factor 2 may correspond to visuospatial orientation and spatial memory, Factor 3 appears to reflect semantic memory and language‐related processes, and Factor 4 represents episodic memory‐related abilities (Table 3 and Figure 2).

**TABLE 3:** Distributions of the 13 retained items in the final 4‐factor model and 3 excluded items from the initial model.

Items	Executive functions (Factor 1)	Spatial memory (Factor 2)	Semantic memory (Factor 3)	Episodic emory (Factor 4)	Excluded
1 Remembering things about family and friends e.g. occupations, birthdays, addresses	—	—	—	—	X
2 Remembering things that have happened recently	—	—	—	X	—
3 Recalling conversations a few days later	—	—	—	X	—
4 Remembering his/her address and telephone number	—	—	X	—	—
5 Remembering what day and month it is	—	—	X	—	—
6‐ Remembering where things are usually kept	—	X	—	—	—
7 Remembering where to find things which have been put in a different place from usual	—	X	—	—	—
8 Knowing how to work familiar machines around the house	—	—	—	—	X
9 Learning to use a new gadget or machine around the house	—	—	—	—	X
10 Learning new things in general	X	—	—	—	—
11 Following a story in a book or on TV	X	—	—	—	—
12 Making decisions on everyday matters	X	—	—	—	—
13 Handling money for shopping	X	—	—	—	—
14 Handling financial matters e.g. the pension, dealing with the bank	X	—	—	—	—
15 Handling other everyday arithmetic problems e.g. knowing how much food to buy, knowing how long between visits from family or friends.	X	—	—	—	—
16 Using his/her intelligence to understand what's going on and to reason things through	X	—	—	—	—

### 3.3 Test‐Retest Reliability

**FIGURE 3:**
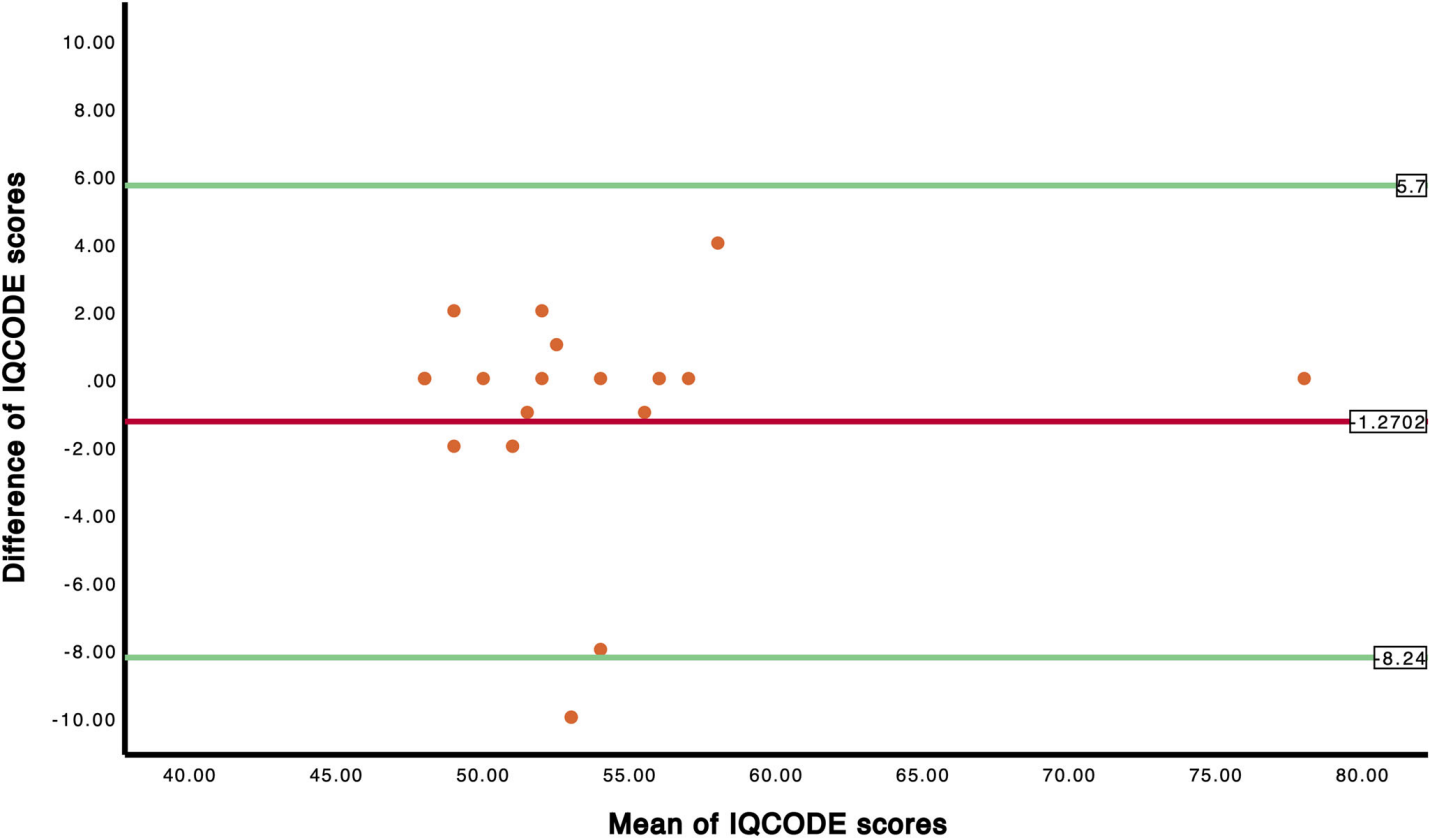
Bland‐Altman plot of IQCODE scores from two different time points of patients.

The IQCODE questionnaire was administered twice to a randomly selected subgroup of 30 patients at a one month interval between assessments to evaluate test‐retest reliability. The clinical and demographical characteristics of the retest group and the main cohort group were well balanced, and no statistically significant differences were observed between the groups (Supplementary Table 1). The Pearson correlation coefficient between these two consecutive measurements was found to be0.83, indicating adequate reliability. Additionally, the Bland‐Altman plot showed no systematic or constant error, and only one value was outside the 95% confidence interval limits (Figure 3).

### 3.4 ROC Analysis

**FIGURE 4:**
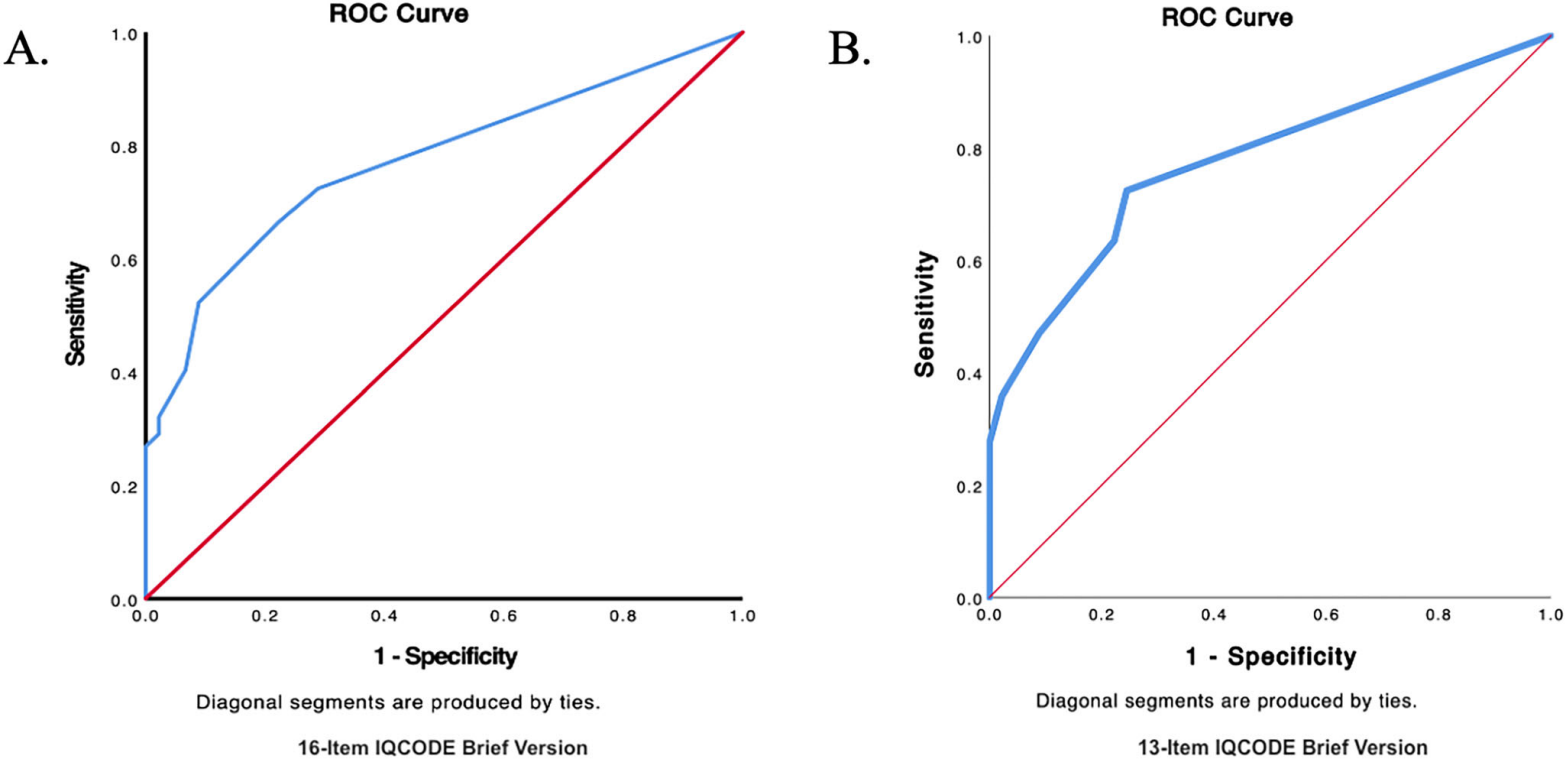
Receiver operating characteristic (ROC) curves of the 16‐item and 13‐item IQCODE brief versions against MoCA‐based cognitive classification. (A) ROC curve for the 16‐item IQCODE brief version (AUC = 0.771; 95% CI, 0.70–0.84), with an optimal cut‐off value of 48.5, sensitivity of 72%, and specificity of 71%; and (B) ROC curve for the 13‐item IQCODE brief version (AUC = 0.775; 95% CI, 0.70–0.84), with an optimal cut‐off value of 39.5, sensitivity of 72%, and specificity of 76%.

Two separate ROC analyses were performed for Model 1 (16 items) and the final model (13 items). For the 16‐item IQCODE brief version, the AUC was 0.771 (95% CI, 0.70–0.84) based on MoCA scores (Figure 4). The optimal cut‐off value was 48.5, yielding a sensitivity of 0.72 and a specificity of 0.71. In the second ROC analysis, the 13‐item IQCODE brief version showed an AUC of 0.775 (95% CI, 0.70–0.84). The optimal cut‐off value for the 13‐item IQCODE brief version was 39.5, with a sensitivity of 0.72 and a specificity of 0.76. Notably, removal of the three items increased specificity from 0.72–0.76.

### 3.5 Sensitivity Analysis

To evaluate the impact of the Minor Heywood case observed for Item 4, an additional EFA was performed after excluding the item. The resulting solution retained a four‐factor structure and explained 74.8% of the total variance, compared with 76.5% in the original model. CFA demonstrated similar model fit (CFI = 0.979, TLI 0.969, RMSEA = 0.068). However, exclusion of Item 4 resulted in one factor being represented by a single item, reducing the conceptual interpretability of the factor structure. Therefore, Item 4 was retained in the final model. Detailed results are provided in (Supplementary Table 6) and 7.

## 4 Discussion

In this study, we demonstrated that the 13‐item IQCODE brief version may be a reliable, clinically useful, and consistent promising screening tool for cognitive impairment in patients with stroke when compared with the in‐person MoCA test. Our findings provide preliminary evidence that the proposed 13‐item IQCODE brief version demonstrated moderate discrimination against MoCA‐based cognitive classification, with a cut‐off value of 39.5 for the 13‐item version yielding a sensitivity of 72% and a specificity of 76%. The 13‐item IQCODE brief version may therefore be useful for identifying cognitive deficits in stroke patients who may require more comprehensive cognitive assessments.

Our work contributes to the limited but growing body of evidence regarding the application of the IQCODE brief version to the stroke population. Previous studies have commonly used the IQCODE brief version to estimate premorbid cognitive status after stroke, but more recent evidence including that of McGovern et al. (2016) indicates that the IQCODE also performs well for detecting PSCI. The majority of the evidence indicates that the IQCODE brief version has primarily been used to assess premorbid cognitive status and the current diagnosis of cognitive impairment, and its ability to predict future dementia remains uncertain. A recent systematic review by Burton et al. (2021) focused on whether the IQCODE can predict future dementia across different patient populations. Only three studies met eligibility criteria, and based on their findings, the authors concluded that there were insufficient data to make any recommendations regarding the use of IQCODE for predicting future dementia.

Although the intended use may differ across disciplines, the IQCODE is a well‐established screening tool that has been translated into several languages over decades. One strength of our study is the re‐evaluation of a widely used test with robust statistical techniques. Factor analysis is a well‐recognized and extensively applied method in the literature (Jöreskog 1969; Schreiber et al. 2006). It not only provides a measure of reliability for a particular scale but also reveals its underlying latent features.

The optimal number of items in the IQCODE has been an ongoing topic in the literature (Reichenheim et al. 2015). Factor analysis may help identify the most appropriate and efficient version of the IQCODE. The development of shorter versions of cognitive screening instruments is often motivated by the need to balance comprehensiveness with feasibility in routine clinical practice. While reducing the number of items inevitably raises concerns regarding content coverage, item reduction may also eliminate the questions that contribute little to the underlying construct being measured. In our study, items 1, 8 and 9 demonstrated weaker or unstable factor loadings and did not contribute consistently to the latent structure identified in stroke survivors. Their exclusion resulted in only a minimal reduction in internal consistency (Cronbach's alpha from 0.95–0.94) while improving factorial coherence and model fit. A minor Heywood case was observed for Item 4. Sensitivity analysis showed that exclusion of item 4 had minimal impact on model fit but resulted in a less interpretable factor structure. Given the small magnitude of the Heywood case and the clinical relevance of the item, it was retained in the final model. Importantly, the remaining 13 items continued to represent a broad range of everyday cognitive abilities relevant to PSCI. Nevertheless, the impact of item reduction on content validity should be further evaluated in independent cohorts and across different clinical populations. PSCI and dementia may affect multiple domains, mainly attention and executive functions (Rost et al. 2022; Weterings et al. 2023). Given these domain‐specific features of post‐stroke dementia, assessing distinct aspects of cognitive function is essential (Rost et al. 2022). In this study, factor analysis revealed various characteristics of the IQCODE brief version, such as spatial and semantic memory components. However, the factor labels should be regarded as heuristic descriptions of item groupings rather than direct representations of discrete neuropsychological domains, since performance in everyday activities typically depends on the interaction of multiple cognitive processes. It should be noted that the identified factors were derived statistically and were not intended to represent independent neuropsychological constructs. The statistically derived factors reflect the patterns of item clustering within the IQCODE, and individual items may involve multiple cognitive processes simultaneously.

On the other hand, stroke survivors are not immune to neurodegenerative dementias; considering the shared risk factors between stroke and Alzheimer's disease, both PSCI and mixed dementia may occur following a stroke (Caratozzolo et al. 2016). Additionally, some patients might have had previous cognitive impairment prior to the stroke, and the stroke could exaggerate the process of neurodegeneration and lead to dementia. Considering that only 18% of patients with mild cognitive impairment patients revert to normal cognition, screening cognitive problems after stroke should be a routine implementation in clinical practice (Canevelli et al. 2016).

Different scoring approaches for the IQCODE have been described in the literature, including the total score and the mean item score. We used the total score, whereas the previous Turkish validation study used the mean‐item score (Ozel‐Kizil et al. 2010). We acknowledge that the use of different scoring methods may limit the comparability of our findings with studies that rely on mean the item score. In that study, the cut‐off value was found to be 3.4 with a sensitivity of 82% and a specificity of 70% for the diagnosis of dementia (AUC 0.855 (95% CI: 0.798–0.912)). The difference between their findings and our results may be explained by two main factors. First, they included patients with different types of dementia, whereas our results are specific to a stroke population. Second, while they used the original 26‐item version, we applied the IQCODE brief version. We also acknowledge that this may limit the comparability of our findings with studies that rely on the total item score.

One strength of this study is its prospective design with uniform data collection. Our study group consisted of prospectively followed stroke patients with an accurate diagnosis, and we did not exclude any subgroup of patients due to their symptoms or lesion localization in the brain. In contrast, patients with aphasia were often excluded from studies because of difficulties in following instructions in previous studies (McGovern et al. 2016). However, post‐stroke aphasia, which is seen in up to 30% of stroke‐survivors, is a common consequence of stroke, and stroke patients with aphasia are equally susceptible to cognitive impairment and dementia (Flowers et al. 2016).

The IQCODE brief version's ability to be administered remotely is another significant benefit. Remote evaluations have been feasible over decades, and telephone interviews are a considerable part of clinical trials and follow‐up evaluations. Screening tests are also used as remote assessment tools; more evidence concerning remote screening tests has been published (Pendlebury et al. 2013; Zietemann et al. 2017). Recently, telephone MoCA (t‐MoCA) was validated in an elderly community‐dwelling cohort, and researchers calculated sensitivity and specificity for the t‐MoCA as 72% and 59%, respectively (Katz et al. 2021). We calculated the 13‐item IQCODE brief version's a sensitivity as 0.72 and specificity as 0.76. Additionally, the brief version of the IQCODE takes about 10 minutes and can be readily administered over the phone or in a remote visit, as we did in our study. Incorporating telephone interviews with cognitive screening into clinical practice may expand the knowledge about PSCI and dementia worldwide since remotely administered screening tools are cost‐effective, well‐tolerated, and allow quick assessment.

In summary, key strengths of this study are the prospective data collection in a standardized manner and consecutive inclusion of stroke patients. Our results showed that an informant‐based questionnaire may be used as a sensitive screening tool for stroke patients in outpatient settings as well. Another distinctive feature of the study was that we evaluated the utility of this questionnaire for validity and reliability analysis using advanced statistical methods, namely factor analysis.

There are several limitations regarding our study. First, the main limitation is that the MoCA test was used for ROC analysis, and the cut‐off value has been defined according to the MoCA results. A comprehensive neuropsychological battery would have been a preferable choice for detecting patients with cognitive impairment and dementia. In addition, a diagnosis of dementia would require further imaging, distinctive biomarkers, and a complete evaluation of the patient. However, one candidate screening test has been assessed against another in this study. Considering the main purpose of our study, this approach was considered a practical and adequate method. Another limitation is the relatively small proportion of hemorrhagic stroke patients in our cohort (10%). Further research on this subgroup is necessary since the severity and pattern of cognitive impairment following hemorrhagic stroke may differ from those following ischemic stroke. The mean age of our study group (58.6 years) is relatively younger compared to the usual stroke population. This may limit the generalizability of the findings to older stroke populations and further studies in older stroke populations are warranted. However, this relatively younger sample also suggests that the observed cognitive deficits were more likely attributable to the stroke itself, reducing the likelihood of pre‐stroke cognitive impairment. Furthermore, while we ensured that informants were close relatives or caregivers, we did not systematically assess informant burden or bias, which could have influenced responses. Variability in informant‐reported assessments may be introduced by caregiver stress and pre‐existing opinions of the patient's cognitive capacity. Finally, both EFA and CFA were conducted using the same dataset, which limits the confirmatory strength of the factorial findings and increases the risk of over fitting. Therefore, the identified 13‐item structure should be considered preliminary until replicated in an independent cohort.

## 5 Conclusion

To our knowledge, this study is the first to show the potential usefulness of a reduced version of the brief IQCODE for cognitive screening in stroke patients, although further validation studies are needed before routine clinical implementation. Early detection of cognitive impairment is essential for appropriate evaluation and management of post‐stroke patients. The brief version of the IQCODE appears to be a feasible, easily applicable, and potentially useful tool for screening cognitive impairment after stroke. However, the proposed 13‐item structure should be considered preliminary until replicated and validated in independent cohorts.

## Author Contributions

**Mine Sezgin**: conceptualization, investigation, Writing – original draft, Writing – review and editing, visualization, validation, methodology, formal analysis, data curation. **Ozan Dörtkol**: data curation, validation, methodology, writing – review and editing. **Nilüfer Yeşilot**: supervision, data curation, project administration, writing – review and editing. **Sevda Özel‐yıldız**: methodology, validation, writing – review and editing, formal analysis, supervision. **Edis Hacılar**: data curation, writing – review and editing, investigation. **Esme Ekizoğlu**: data curation, investigation, writing – review and editing.

## Funding

The authors have nothing to report.

## Ethics Statement

This study protocol was reviewed and approved by Istanbul University Ethic Committee (protocol number: 2023/315).

## Consent

Written informed consent was obtained from all participants (or their parent/legal guardian/next of kin) to participate in the study.

## AI Declaration

Artificial intelligence tools were used exclusively for language editing and grammatical refinement during the preparation of the manuscript. No AI tools were used for data analysis, interpretation of results, or manuscript content generation.

## Conflicts of Interest

The authors declare no conflicts of interest.

## Supporting information




**Supplementary Material**: brb371649‐sup‐0001‐TableS1‐S7.docx

## Data Availability

The datasets generated during and/or analyzed during the current study are available from the corresponding author on reasonable request.
