# Supplementary material for: IQCODE‐Brief Version in Stroke Patients
Source: Brain Behav. 2026 Aug 2;16(8):e71649. doi: 10.1002/brb3.71649 (PMC13430275; doi:10.1002/brb3.71649)
Supplement: Supplementary file 1 — Supplementary Material: brb371649‐sup‐0001‐TableS1‐S7.docx [file BRB3-16-e71649-s001.docx]

**IQCODE-BRIEF VERSION IN STROKE PATIENTS**

Mine Sezgin^a,b^, Edis Hacılar^a^, Sevda Özel-Yıldız^c^, Ozan Dörtkol^a^, Esme Ekizoğlu^a^, Nilüfer Yeşilot^a^

^a^ İstanbul University Istanbul Faculty of Medicine, Department of Neurology, İstanbul, Türkiye

^b^ Istanbul University, Department of Biostatistics, Institute of Graduate Studies in Health Sciences, Istanbul, Türkiye

^c^ İstanbul University Istanbul Faculty of Medicine, Department of Biostatistics, İstanbul, Türkiye

**Corresponding Author:** Mine Sezgin, MD

Istanbul University, Istanbul Medical Faculty

Department of Neurology

34390 Çapa İstanbul/ Türkiye

Phone: +90-212-414-20-00- 30225

e-mail ; [mine.sezgin@istanbul.edu.tr](mailto:mine.sezgin@istanbul.edu.tr) ; [szgnmn@gmail.com](mailto:szgnmn@gmail.com)

|  | Retest group  (n=30) | Main group  (n=149) | p |
| --- | --- | --- | --- |
| Gender (male, n, (%)) | 22, (73.3) | 91 (61.1) | 0.222ǂ |
| Age (year, mean (SD); median IQR) | 59.3 ±15.6;  63 (49-71) | 58.8 ±13.4;  58 (51-68) | 0.595^#^ |
| Education (years, mean (SD); median IQR) | 8.2 ±4.3;  8 (5-12) | 8.85 ±4.5;  8 (5-12) | 0.751^#^ |
| Hypertension, n, (%) | 21, (72.4) | 114, (78.6) | 0.470ǂ |
| Diabetes, n, (%) | 4, (13.8) | 53, (36.6) | 0.017ǂ |
| Atrial fibrillation, n, (%) | 2, (6.9) | 22, (15.2) | 0.376ǂ |
| Ischemic stroke, n, (%) | 27 (90.0) | 134 (89.9) | 0.991ǂ |
| Lesion localization, n, (%)  Supratentorial  Right  Bilateral | 18, (62.1)  13, (44.8)  4, (13.8) | 94, (63.1)  53, (35.6)  20, (13.4) | 0.884ǂ  0.601ǂ |
| NIHSS at index stroke (mean (SD); median IQR) | 5.0 ±5.3;  3 (1.7-6.2) | 4.9 ±5.3;  4 (2-6) | 0.883^#^ |
| mRS≥3 at index stroke n, (%) | 18, (62.1) | 90, (62.5) | 0.149ǂ |
| NIHSS (at last control: mean (SD); median IQR) | 1.2 ±1.6;  1 (0-2) | 1.1 ±1.4;  1(0-2) | 0.863^#^ |
| Total MoCA score (mean (SD); median IQR) | 19.2 ±5.6;  20 (16-23) | 19.8 ±6.03;  21 (16-25) | 0.489^#^ |
| Total IQCODE brief form score (mean (SD); median IQR | 52.7 ±6.66;  50 (48-55) | 51.7 ±5.84;  50 (48-52) | 0.375^#^ |

**Supplementary Table 1**. Comparison of the clinical and demographic characteristics between the retest group and the main group

ǂ Chi-square test; ^#^Mann-Whitney U test

n: number; SD: standard deviation; IQR: inter quartile range, NIHSS: National Institutes of Health Stroke Scale; mRS: modified Rankin scale; MoCA: Montreal Cognitive Assessment test, IQCODE: The Informant Questionnaire on Cognitive Decline in the Elderly

**Supplementary Table 2.** Uniqueness and communality values of the questionnaire items

| Items | Uniqueness | Communality |
| --- | --- | --- |
| Item-2 | 0.4497 | 0.5503 |
| Item-3 | 0.0896 | 0.9104 |
| Item-4 | -0.0008 | 1.0008 |
| Item-5 | 0.1773 | 0.8227 |
| Item-6 | 0.1256 | 0.8744 |
| Item-7 | 0.1984 | 0.8016 |
| Item-10 | 0.3214 | 0.6786 |
| Item-11 | 0.3187 | 0.6813 |
| Item-12 | 0.3240 | 0.6760 |
| Item-13 | 0.2192 | 0.7808 |
| Item-14 | 0.2363 | 0.7637 |
| Item-15 | 0.2407 | 0.7593 |
| Item-16 | 0.3506 | 0.6494 |

**Supplementary Table 3.** Secondary factor loadings (loading threshold set at 0.10)

|  | Factor | | | |  |
| --- | --- | --- | --- | --- | --- |
|  | 1 | 2 | 3 | 4 | Uniqueness |
| Item 2 | 0.285 |  |  | 0.539 | 0.4497 |
| Item 3 |  |  |  | 0.938 | 0.0896 |
| Item 4 |  |  | 1.017 |  | -8.06e-4 |
| Item 5 | 0.185 |  | 0.670 |  | 0.1773 |
| Item 6 |  | 0.964 |  |  | 0.1256 |
| Item 7 |  | 0.809 | 0.107 |  | 0.1984 |
| Item 10 | 0.601 | 0.197 |  | 0.122 | 0.3214 |
| Item 11 | 0.831 |  |  |  | 0.3187 |
| Item 12 | 0.735 | 0.117 |  |  | 0.3240 |
| Item 13 | 0.845 |  | 0.146 |  | 0.2192 |
| Item 14 | 0.861 |  |  |  | 0.2363 |
| Item 15 | 0.913 |  |  |  | 0.2407 |
| Item 16 | 0.658 |  |  | 0.203 | 0.3506 |

Standardized pattern matrix coefficients obtained using the minimum residual extraction method with oblimin rotation are presented

**Supplementary Table 4**. Inter-factor correlations

|  | 1 | 2 | 3 | 4 |
| --- | --- | --- | --- | --- |
| 1 | - | 0.527 | 0.632 | 0.624 |
| 2 |  | - | 0.417 | 0.480 |
| 3 |  |  | - | 0.507 |
| 4 |  |  |  | - |

**Supplementary Table 5.** Reliability indices for each factor;

| Factors | Cronbach’s alpha |
| --- | --- |
| Factor 1 | 0.938 |
| Factor 2 | 0.900 |
| Factor 3 | 0.932 |
| Factor 4 | 0.803 |

**Supplementary Table 6.** Sensitivity analysis excluding Item 4

| Model | CFI | TLI | RMSEA |
| --- | --- | --- | --- |
| Original model | 0.973 | 0.964 | 0.070 |
| Excluding Item 4 | 0.979 | 0.969 | 0.068 |

CFI: Comparative fit index TLI: Tucker Lewis index, RMSEA: The Root Mean Square Error of Approximation

**Supplementary Table 7.** Factor loadings after excluding Item 4

|  | Factor | | | |  |
| --- | --- | --- | --- | --- | --- |
|  | 1 | 2 | 3 | 4 | Uniqueness |
| Item 2 | 0.430 |  | 0.497 |  | 0.44379 |
| Item 3 |  |  | 0.966 |  | 0.04434 |
| Item 5 |  |  |  |  | 0.42021 |
| Item 6 |  | 0.918 |  |  | 0.21168 |
| Item 7 |  | 0.898 |  |  | 0.13577 |
| Item 10 | 0.585 |  |  |  | 0.31311 |
| Item 11 | 0.702 |  |  |  | 0.32024 |
| Item 12 | 0.538 |  |  |  | 0.32995 |
| Item 13 |  |  |  | 0.941 | 0.00246 |
| Item 14 | 0.598 |  |  |  | 0.24688 |
| Item 15 | 0.936 |  |  |  | 0.19704 |
| Item 16 | 0.408 |  |  |  | 0.35467 |

Item 4 was excluded from the final 13-item model and the EFA was repeated using the same extraction method (minimum residual) and oblimin rotation with a fixed four-factor solution.
